# Enhancing post-training evaluation of annual performance agreement training: A fusion of fsQCA and artificial neural network approach

**DOI:** 10.1371/journal.pone.0305916

**Published:** 2024-06-25

**Authors:** Md. Zohurul Islam, Munshi Muhammad Abdul Kader Jilani, Mohammad Rezaul Karim

**Affiliations:** 1 Research and Development Department, Bangladesh Public Administration Training Centre (BPATC), Savar, Dhaka, Bangladesh; 2 Department of Human Resource Management, Bangladesh Institute of Governance and Management (BIGM), University of Dhaka (Affiliated), Dhaka, Bangladesh; 3 Department of Governmental System, Bangladesh Public Administration Training Centre (BPATC), Savar, Dhaka, Bangladesh; University of Education, Lahore, PAKISTAN

## Abstract

This study aims to enhance the post-training evaluation of the annual performance agreement (APA) training organized by the Bangladesh Public Administration Training Centre (BPATC), the apex training institute for civil servants. Utilizing fuzzy-set qualitative comparative analysis (fsQCA) and artificial neural network (ANN) techniques within Kirkpatrick’s four-stage model framework, data were collected from a self-administered questionnaire survey of 71 in-service civil servants who participated in the APA training program. This study employs an asymmetric, non-linear model analyzed through a configurational approach and ANN to explore interrelationships among the four Kirkpatrick levels namely, reaction, learning, behavior, and results. Findings indicate that trainees were satisfied across all levels, identifying a non-linear relationship among these levels in post-training evaluation process. The research highlights that "learning skills" are most significant in the APA post-training evaluation, followed by behavior, results, and reaction. Theoretically, this research advances Kirkpatrick’s model and adds to the literature on public service post-training evaluation. Practically, it recommends prioritizing strategies that address cognitive barriers to enhance training effectiveness. This study’s innovative approach lies in its concurrent use of fsQCA and ANN methods to analyze the success or failure of APA-related trainees, offering alternative pathways to desired outcomes and contrasting traditional quantitative methods that provide a single solution. The findings have practical implications for public service training institutions and bureaucratic policymakers involved in capacity development, guiding the creation of more effective in-service training courses for public officials. The methodology and analysis can be applied in other contexts, allowing bureaucratic policymakers to replicate these findings in their learning institutes to identify unique configurations that lead to successful or unsuccessful training outcomes, adopt effective strategies, and avoid detrimental ones.

## Introduction

In the contemporary era marked by rapid transformations [1], the significance of an efficient, competent, and adaptable public service has become increasingly the highest priority [2]. This is also crucial in order to effectively provide value and cultivate public trust in governmental entities. In Bangladesh, the strategic framework of Vision 2041 and its corresponding policy document Perspective Plan (PP 2041) are founded upon four vital institutional pillars. Notably, the fourth pillar of Vision 2041 places significant emphasis on the critical aspect of strengthening the capacity of public officials through training [2]. To enhance the efficacy to accelerate public service and increase its effectiveness, the Government of Bangladesh (GoB) established the Public Administration Reform Commission (PARC) in 1997 [3]. In its study [4], the PARC recognized that the performance of an organization within a specific timeframe is a critical determinant of its success [5, 6]. Moreover, the characterization of an organization’s economic and exterior results at a specific time relies upon the utilization of preset performance metrics to allocate budget. Meanwhile, in 2009, GoB enacted the Budget Management Act, which simultaneously signaled the beginning of performance management. Therefore, enhancing civil servants and public officials’ abilities aligns them with a dynamic economy emphasizing collaborative efforts, capacity growth, and internal governance and functioning [7, 8]. In light of several activities, GoB initiated the Performance Management System (PMS) under the Annual Performance Agreement (APA) in the fiscal year 2014–15, specifically at ministry/division levels. In the initial phase, the government intended a strategy to disseminate the plan throughout all levels of the organizations, including the lowest tier. As a result, the extension was implemented at the department level during the year 2015–16 and subsequently at the field level in the year 2016–17 [9].

The Bangladesh Public Administration Training Centre (hereafter BPATC), recognized as the leading training institution in the public sector, has been entrusted with coordinating training initiatives focused on the APA. The main intention is to provide training on the APA to enhance civil servants’ skills and capabilities. West [10] exposed that the APA is an influential instrument that can have a crucial impact on promoting transparency, accountability, and adaptability within public institutions. Furthermore, implementing the ’Annual Performance Agreement’ can significantly enhance the accountability and effectiveness of the governing bodies inside their particular entities [9]. Consequently, BPATC implemented a series of training initiatives aimed to enhance the skills and knowledge of public sector officials across various hierarchical positions, ranging from entry-level to senior-level positions. As stipulated in terms of the training impact, BPATC was required to undertake a research study on the post-training evaluation of a designated training program. The present study has been devised to assess the effectiveness of the training program on the APA guidelines, which has been previously provided at various public sector officials.

Post-training evaluation serves as a tool to gauge the effectiveness and practicality of the training session for the trainees. Because training efficacy is influenced by various aspects, as indicated by previous scholarly research. The amalgamation of learners’ skills, aptitude and motivation has been identified as a key determinant of training effectiveness [11, 12]. It can be inferred that the performance of APA training programs will likely improve when trainees possess both motivation and are prepared to acquire knowledge [13, 14]. Notably, individual attributes, especially self-efficacy and learning motivation, have a significant role in determining the effectiveness of training programs.

Trivedi [15] mentioned that performance management system (PMS) can be manifested in a number of different ways, with the performance contract system being one of the most accepted. The GoB opted for this one, which described an APA as a "record of comprehension between a Minister who represents the Mandate of the Citizen, and the Secretary of Ministry or Division entrusted with carrying out /executing this Mandate." [16]. However, the reality is that the APA is an agreement of establishing a connection between the political and administrative leaders underscores its instrumental significance as a framework intended to translate political aspirations and objectives into actionable administration strategies. This perspective asserts that the APA facilitates and encourages communication and interaction between the bureaucracy and citizens. Likewise, the ministries are responsible for planning, prioritizing, and selecting their most significant undertakings within a particular fiscal year, exerting their utmost efforts to execute plans successfully. [16].

While numerous studies [17–19] have been executed to examine the extent to which developing nations have adopted the APA principles, there has been a lack of focus on exploring the evaluation of post-training APA performance when applying a performance-based framework. This study aims to address this research gap by conducting a configurational and artificial neural network (ANN) analysis of the effectiveness of post-training APA training in a particular developing nation, namely Bangladesh. In 2014, the GoB officially introduced APA as an essential accountability measure based on performance evaluation. The ultimate goal of APA was to implement a management and accountability structure that is centered on evaluation criteria. There is a dearth of configurational as well as ANN analytical studies undertaken to evaluate the level of accomplishment achieved by the novel reform endeavor in Bangladesh Public Administration. This paper uncovers the effectiveness of post-training evaluation on the APA initiative in promoting transparency and accountability of public officials, civil servants in particular.

The evaluation phase concludes the training outcomes and is of utmost importance in assessing the effectiveness of the training. Nevertheless, this aspect is frequently overlooked or given less priority [20]. Even though, the assessment of the training program is an essential component in evaluating the effectiveness of the training course. However, training institutions often encounter challenges in comprehending the diverse methodologies, procedures, and evidentiary criteria associated with conducting evaluations, leading to dissatisfaction [21] and addressed by Alsalamah and Callinan [22]. The insufficient and erratically implemented evaluation system could potentially impede the efficacy of training. Importantly, inadequate knowledge or limited availability of evaluation methodologies and instruments may contribute to substandard evaluation practices. Nevertheless, it is unnecessary for the assessment process to be excessively intricate.

This article presents an analytical and pragmatic strategy for assessing the asymmetrical analysis of the post-training evaluation of the APA training organized for government officials in Bangladesh. The approach involves adapting Kirkpatrick’s four-level model of training criteria, a widely utilized framework for evaluating training in business institutions for evaluation training initially published in 1959. It is evident that there are several methods available for evaluating training programs, including ROI [23], cost-benefit analysis [24], CIPP (context, input, process, and product) mentioned by Badrujaman, Arthur [25], CIRO technique [26], and others. However, the Kirkpatrick model is particularly suitable for assessing both individual and organizational perspectives.

Fuzzy-set qualitative comparative analysis (fsQCA) is a pioneering technique [27] used to investigate causal relationships. It provides path configurations that allow for the identification of various options (solutions) that can account for a similar outcome. In line with this, fsQCA is employed to analyze how ’Kirkpatrick’s Four-Level Evaluation’ criteria among public service officials are influenced by their antecedents in this research. Pappas, Mikalef [28] argued that fsQCA offers a comprehensive and robust interpretation of the data, serving as an alternative approach to conventional variance-based techniques. The results of fsQCA suggest various, distinct and equally potent combinations of reaction, learning abilities, behavior, and result (APA oriented performance) that elucidate for the success of APA training after it has been completed.

This research enhances the existing literature in three key aspects. Firstly, it addresses a significant gap by conducting an in-depth analysis that leverages fsQCA and ANN methodologies. This approach offers a detailed understanding of the complex interactions and synergies within the Kirkpatrick Model. Consequently, the research fills identified voids and introduces new perspectives on the interplay of these factors within the established framework. Secondly, the study applies Kirkpatrick’s four-level evaluation criteria—reaction, learning, behavior, and results—to examine participants’ opinions of the APA training. It also innovates by using fsQCA to analyze the causal links between trainees’ views on the effectiveness of post-APA training. Uncovering these constructs can help trainers and practitioners identify what motivates trainees, leading to improved training content and refined APA curriculum or online training techniques.

The structure of the paper is such that the following section offers a theoretical overview of the model, succeeded by a review of the relevant literature. The research questions are also presented, and research variables are later operationalized. Afterwards, the third section of this paper elucidates the formulation of hypotheses and provides a comprehensive account of the methodology employed in our study. The fourth component of this study provides an analysis and interpretation of the data, followed by an extensive discussion of their implications. The last segment of the paper examines the conclusions, limitations, and potential avenues for future study in post-training APA evaluation.

## Background and hypothesis development

### Theoretical model

**Fig 1:**
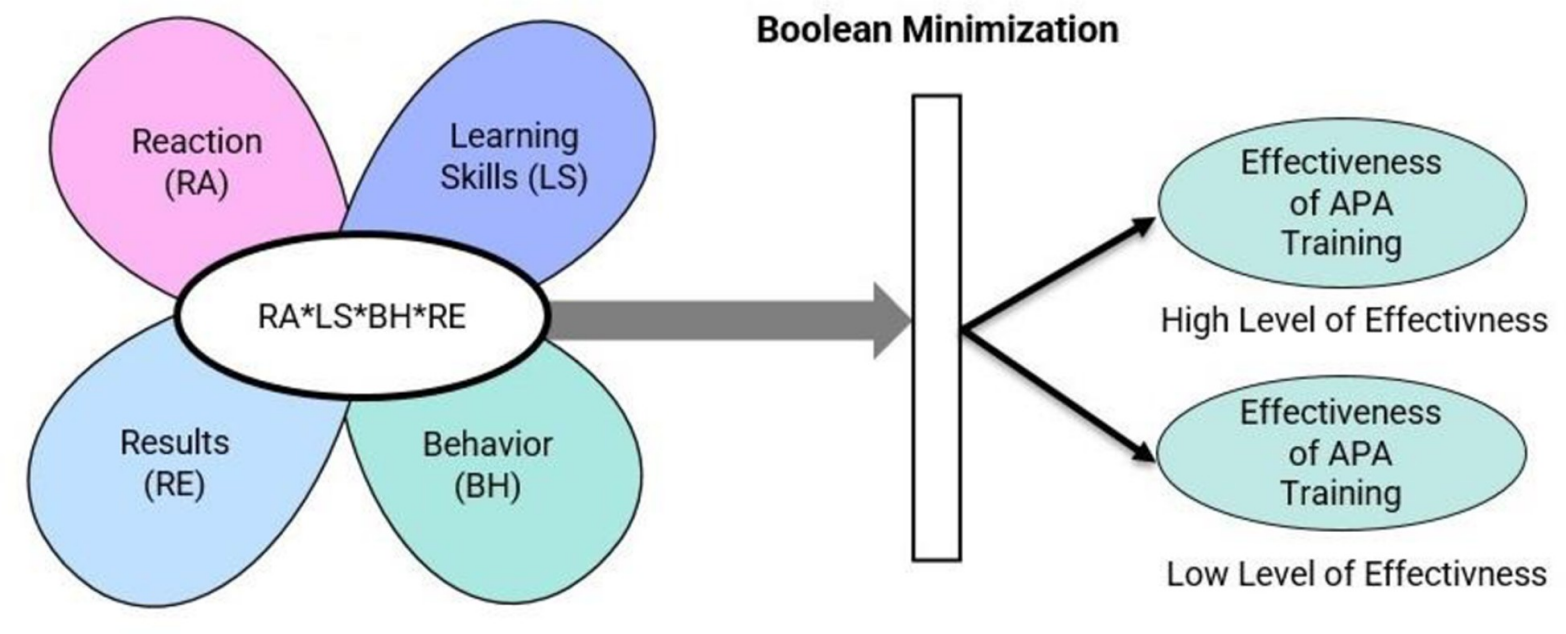
Conceptual framework based on fsQCA.

Kirkpatrick’s evaluation model, proposed in 1959, remains a widely cited and well-regarded paradigm and classical framework for assessing training programs [29]. Despite an augmented version by Alliger, Tannenbaum [30], the original model prevails in usage. Its appeal lies in its ability to demonstrate training results through different data at various evaluation levels. The four levels of Kirkpatrick’s evaluation model [31] are (1) Reaction, (2) Learning, (3) Behavior, and (4) Result, and it assesses both formal and informal training methods and rates them against four levels [32]. While extensively applied in the training and development sector, its use in the APA domain is limited. Despite calls from researchers, its applicability in evaluating APA training is an area that has yet to be extensively explored [8, 19, 26]. Moreover, the Kirkpatrick model’s simplicity, practicality, and ease of comprehension make it a readily accessible [33] and structurally sound approach for post-training evaluation. The model is also recognized for its efficiency, as it does not demand excessive time from the administrator [34]. Notably, the Kirkpatrick model has significantly contributed to the theoretical understanding of evaluation and has played a prominent role in shaping evaluative practices [35]. Its prevalence in academic research is evidenced by its high citation rates [36]. Drawing on this model, the conceptual framework for this study is illustrated in Fig 1.

While scholars such as Holton III [37] and Zevin, Morkem [38] have criticized the model’s hierarchical structure, existing studies and empirical findings do not provide adequate support for the presumption of its hierarchical organization. Moreover, there have been critiques directed against the accuracy of this model, specifically about the assumed cause-and-effect relationship between its levels and the importance assigned to the progressive evolution of learning outcomes. Nevertheless, Kirkpatrick and Kirkpatrick [39] contend that the levels of their model can be measured in any sequence without necessitating an assumption of causality. As many researchers and practitioners confirm the Kirkpatrick model’s ongoing utility, usefulness and effectiveness, the model continues to be widely used even in the face of criticism [36, 40]. Nonetheless, a number of models like the CIRO model by Warr, Bird [41], Philips’s ROI model by Phillips [40], Brinkerhooffs success case method by Brinkerhoff [42], CIPP model by Stufflebeam [43] were put out to assess post-training. As Tamkin, Yarnall [44] and Bove and Little [45] noted, several assessment models are essentially Kirkpatrick’s model extensions. These models integrate substantial components from the original framework and build upon it, typically making improvements either at the beginning, such as integrating training design or needs analysis, or later on, by introducing evaluations of overall results. In some instances, these models are expanded by adding adjustments at the beginning and end of the evaluation procedure. Numerous models have added an evaluation of return on investment, broadening Kirkpatrick’s framework. However, it is worth noting that this aspect can be covered under the ‘Results level’, which is Level 4. Consequently, the majority of the assessment models documented in the literature are derived from the Kirkpatrick model [36, 46]. Even so, nearly every one of current training assessment methods are too intricate, expensive to execute, and time-intensive [32, 36].

### Training evaluation and effectiveness

Training is a systematic learning process aimed at creating sustainable transformations in an individual’s knowledge, attitudes, and abilities [14]. Its importance is underlined in both the perspective of employees and employers since it not only enhances present task performance but also enables employees to participate in more innovative and intricate tasks. As a result, training opens new avenues for greater career development, thereby enhancing employees’ competitiveness in the workplace [47]. The effectiveness of training is a multifaceted concept, incorporating both immediate and long-term outcomes. Bushnell [48] posited training effectiveness as a blend of short-term outputs and long-term results. It mostly involves assessing the learning progress of trainees and their subsequent enhancements in job performance. Training effectiveness evaluation is a systematic process designed to collect and analyze outcome data, serving a dual purpose such as assessing whether training objectives have been met and enhancing the overall training quality [14]. In this sense, Kirkpatrick’s model addressed by Kucherov and Manokhina [47] four-level framework is instrumental in evaluating this APA training, divided into Reaction, which refers to the immediate responses of trainees; Learning, which measures the knowledge or skill gained through training; Behaviour, which assesses the application of acquired skills in the workplace; and Results, which evaluates the impact of the training on organizational goals. This model offers a sophisticated and comprehensive approach for assessing post-training effectiveness, blending both qualitative and quantitative aspects.

In the academic literature features a wide array of definitions for post-training evaluation, each concentrating on a particular facet of the evaluation’s goal and methodology. Brown and Sitzmann [49] conceptualize it as an evaluation process for ascertaining the effectiveness and efficiency of instructional programs, emphasizing its role in gauging training success against its objectives. Other perspectives highlight its feedback role, defining it as an effort to collect data on a training program’s impact to assess its value [26]. Additionally, it is defined as a methodical strategy for collecting and analyzing training-related data to assess the efficiency and effectiveness of training interventions [36, 49]. These definitions collectively enrich the understanding of training evaluation, highlighting its crucial role in refining the quality and effectiveness of learning and professional training efforts.

### Reaction

The ’Reaction’ level in training evaluation largely assesses the subjective impressions and attitudes of the trainees towards the training program. Typically, it is evaluated by end-of-course assessment questionnaires, which are a commonly used way to obtain immediate feedback on the content and delivery of the training [50, 51]. The trainees’ reactions operate as initial indications of the effectiveness of the APA training, with positive reactions often being associated with increased engagement, motivation, and learning outcomes. In contrast, adverse responses can bring attention to problems about the pertinence of the material, how it is presented, or the methods used to engage the audience, which could potentially affect its effectiveness [26]. Hence, examining the trainees’ reactions to assess their emotional and attitudinal responses is crucial, which can offer notable insight for boosting future training efforts. Therefore, the analysis suggests the following hypothesis:

H1: Trainees’ reactions significantly correlate with the effectiveness of APA training.

### Learning

The ’Learning’ level in post-training evaluation assesses how much participants/trainees have changed their attitudes, enhanced their knowledge, and gained new skills following the capacity-building training [39]. This level employs quantifiable measures to determine the extent of learning achieved, typically by participants’ self-assessment of their progress in learning [35, 47]. The classification of learning outcomes can be divided into three distinct categories, i.e., cognitive, skill-based, and attitudinal. Cognitive outcomes pertain to knowledge acquisition, skill-based effects concentrate on developing technical skills, and attitudinal outcomes involve changes in goals, motivation, and attitudes that are aligned with the aims of the training [52]. The participants’ increased knowledge, changed attitudes, and enhanced skills acquired through training are shreds of evidence of successful learning [53]. In essence, the results of the ’Learning’ level are closely tied to the effectiveness of APA training. These outcomes represent the measurable and intangible benefits gained by trainees, which in turn contribute to the success of the training program. Based on the preceding discussion, the study posits the following hypothesis:

H2: Trainees’ learning skills positively correlate with the effectiveness of APA training.

### Behavioral

The ’Behavior’ level in training assessment evaluates the practical use of learned information and skills in the work environment. For a training program to be deemed effective, it is crucial that participants reflect favorable changes in their behavior and successfully apply what they acquired knowledge to their employment duties [53]. Assessing this transfer of knowledge is complex and cannot be comprehensively captured alone through quantitative approaches. Besides this, Kirkpatrick suggests combining quantitative and qualitative methods to better understand participants’ behavior after training [22, 48]. Kirkpatrick also recommends that the optimal timeframe for assessing behavioral modifications is at least three months after training. This acknowledges that these changes may require up to six months to become firmly established or may vary over time [54]. Corroborating this, Axtell, Maitlis [55] discovered that the degree of knowledge transfer one month following training is a robust predictor of the level of transfer one year later. This approach recognizes the complex and fluctuating nature of human behavior in relation to APA training, highlighting the necessity for a well-defined and diverse assessment strategy that considers both time constraints and topological approaches to effectively measure the effectiveness of the training. Thus, the prior discussion indicates a positive link between trainees’ reactions and the success of APA training. The research postulates, in light of the foregoing, that

H3: Trainees’ behavior positively enhances the effectiveness of APA training.

### Results

The ’Results’ stage of the Kirkpatrick Model, which is crucial in evaluating training, focuses on assessing the influence of training on both organizational performance and the performance of individual trainees, specifically in enhancing job performance [39]. Assessing this level is highly intricate due to multiple challenges, such as the trainees’ awareness of the evaluation process, the time lapse between finishing the training and assessing performance, and external influences on the training’s effectiveness [25]. The assessment approach in the public sector must be customized to align with the unique service delivery environment, stakeholders’ requirements, and outcomes, which are distinct from those in the commercial sector [19]. Unlike business contexts that prioritize financial gains, the impact of training in the public sector cannot be quantified in monetary terms [2]. In order to successfully evaluate training results, it is necessary to take into account the viewpoints of different stakeholders. The study evaluates the effectiveness of APA training among public servants, emphasizing the significance of improving leadership abilities within the APA framework. The link between the ’Results’ element and training effectiveness is vital. The assessment evaluates how the training achieves its intended goals, including enhancing productivity, improving work quality, reducing costs, fostering growth, and increasing satisfaction [8]. The ability of training to successfully lead to favorable organizational transformations serves as evidence of its effectiveness. The link indicates that when training is effective, meaning it is well received and absorbed by participants, it results in the desired outcomes for the organization as a whole. This result highlights the significance of an accurate assessment process that takes into account various viewpoints from stakeholders, particularly in the public sector. Based on the previously mentioned arguments, the study postulates that

H4: Trainees’ results (performance) positively improve the effectiveness of APA training.

## Research method

### Qualitative comparative analysis and fsQCA

Based on case comparison standards and set-theoretic Boolean algebra logic, qualitative comparative analysis (QCA) is a highly regarded study technique used mostly in qualitative research to evaluate theoretical hypotheses and produce insights [56]. It is viable to find combinations or configurations of factors, drivers, and antecedents that result in particular outcomes by employing QCA [57]. Bernardino and Curado [50] and Sánchez-Mena, Martí-Parreño [58] argue that in areas like training evaluation in public sector research and higher education training, an outcome is better understood as a configuration of interrelated structures rather than a separate, isolated object. In addition to being particularly helpful for creating, elaborating, and testing theories, QCA can be applied to inductive, deductive, and abductive reasoning as well, as addressed by Park, Fiss [59], whereas Sánchez-Mena, Martí-Parreño [58] and Bernardino and Curado [50] have all shown how to use and modify it for training assessment.

Fuzzy-set QCA is a method that combines qualitative and quantitative assessments to ascertain the extent to which a case belongs to a particular category [60]. This methodology provides flexibility and uncovers combinations of factors/elements that traditional variance-based methods may fail to consider [61]. Unlike approaches that rely on variance, fsQCA examines numerous combinations of criteria by dividing the sample into subgroups, thus accommodating a range of solutions. Its flexibility stems from not being subject to exceptions, making it valuable for capturing both significant and nuanced aspects of the sample [62]. Nevertheless, deliberate choices and thorough documentation are essential for ensuring the research’s study effectiveness and the analysis’s reliability.

### Research design

The present study employs a survey to collect data from a specific sample, investigating the relationships between relevant aspects of the research. The survey is administered online, utilizing a standardized survey questionnaire to gather responses to minimize errors in data collection. The responses were collected solely from public officials who participated in the APA training conducted by BPATC. Furthermore, the individuals involved were government officials working in several areas of public administration at both the national and regional levels. This study examines explicitly the evaluation that takes place after training, using the Kirkpatrick model as a tool to measure the effectiveness of the training program. The study variables were obtained from this model, including reaction, learning, behavior, and results, along with the participants’ demographic data.

### Data collection and survey instruments

We employ a multidimensional strategy to evaluate post-training effectiveness within APA training, incorporating components derived from the Kirkpatrick model’s four levels as independent variables. These levels, encompassing reaction, learning, behavior, and results, serve as vital elements for evaluation, with the training effectiveness of APA being the dependent variable. Previous studies [22, 50] examining the assessment of different training programs and individual performance provide the foundation for the reasoning behind the selection of these dimensions. This research employed a structured questionnaire developed based on Kirkpatrick’s four-level evaluation model [39], existing literature, and insights from previous studies on training effectiveness [14]. To ensure the validity of the questionnaire, a panel of four experts was assembled, comprising an academic, an executive director from a training organization, a secretary with expertise in the APA training process, and a senior APA trainer. These experts conducted thorough reviews to assess the questionnaire’s applicability, coverage and correctness. Additionally, a qualitative review involving trainees with a minimum of six months of post-APA training experience was conducted to refine the questionnaire items further. Following feedback and reviews, three items were eliminated to enhance completeness and accuracy. The subsequent entries have been morphologically modified. The questionnaire comprises three sections: demographic information about trainees, the four elements of the Kirkpatrick Model, and the training effectiveness of APA. The final questionnaire included 21 items, with five assessing the training effectiveness of APA. Likewise, four questions gauged reactions, four assessed learning skills, four measured behaviors, and the remaining four examined results. A five-point Likert scale ranging from 1 ("strongly disagree") to 5 ("strongly agree") was utilized (Details questionnaire in S1 Table). Participants were asked to choose a scale reflecting their comprehension, experience, and knowledge regarding each questionnaire item. From July 1, 2022 to September 30, 2022 data were gathered for the post-training evaluation on APA training.

### Sample and data analytical process

The sample comprises government officials from several ministries in Bangladesh. Trainees who attended APA training at BPATC during the epidemic were invited to participate in the survey via email. The survey employed a five-point Likert scale ranging from 1, indicating "strongly disagree," to 5, indicating "strongly agree." Of the 84 questionnaires put forward, only 71 replies were included in the final analysis. The remaining 13 responses were deemed invalid because of problems such as infidelity and missing information. Fraenkel, Wallen [63] preferred this approach when selecting the sample because it showed that the participants shared a high degree of similarity in their fundamental occupational inclination. Various statistical methods were employed to test hypotheses and reach conclusive research findings, including descriptive statistics, reliability and correlation analysis, contrarian case analysis, fsQCA, and ANN.

## Data analytics and results

### Demographic profile

This study intended to collect data from 71 government officials who received APA training from BPATC and were affiliated with various public organizations in Bangladesh. The demographic details of the participants, as outlined in Table 1, included job position, gender, age, marital status, educational qualification, and years of service. It is worth noting that the majority of participants were associated with public organizations and held a wide range of roles. For example, 47.90% were assistant secretaries, 7.00% were deputy secretaries, 18.13% were senior assistant secretaries, and 26.28% held other positions. The majority of respondents were male (84.05%), and the age distribution was categorized accordingly. In terms of academic qualifications, 63.40% held master’s degrees, 28.20% had bachelor’s degrees, 5.60% possessed doctoral degrees, and 2.80% held other professional degrees. Additionally, the level of experience varied among respondents, with 53.5, 23.9, 2.8, 9.9, 2.8, and 7.0% having less than 5, 5–10, 11–15, 16–20, 21–25, and more than 25 years of experience, respectively.

**Table 1:** Demographic characteristics of the APA training received.

Trainee Categories	Number (s)	Percentage (%)
*Position(s)*		
Assistant Secretary/Equivalent	34	47.90
Deputy Secretary/Equivalent	05	7.00
Senior Assistant Secretary	13	18.13
Others	19	26.80
*Gender*		
Male	60	84.05
Female	11	15.50
*Marital Status*		
Married	61	85.90
Unmarried	10	14.10
*Respondents Age*		
25–30 years	17	23.90
31–35 years	32	45.10
36–40 years	06	8.50
41–45 years	04	5.60
46–50 years	06	8.50
51 years and above	06	8.50
*Academic Degree*		
Bachelor	20	28.20
Masters	45	63.40
PhD	04	5.60
Others	02	2.80
*Service Length (Year)*		
Less than 5	38	53.50
5 to 10 years	17	23.90
11 to 15 years	2	2.8
16 to 20 years	7	9.9
21 to 25 years	2	2.8
25 years and more	5	7.0

### Reliability and validity

Reliability, as an indicator of result stability over time, was assessed using Cronbach’s **α**, with a value exceeding 0.80 (0.856) across all five constructs—reaction, learning skills, behavior, results, and effectiveness of APA training—signifying strong internal consistency [64]. Each dimension demonstrated satisfactory reliability, with Cronbach’s α values surpassing 0.80 (.939, .960, .965, .856, and .910, respectively), as shown in Table 2. Furthermore, multicollinearity was evaluated through variance inflation factor (VIF) and tolerance tests, with criteria set at VIF < 10 and tolerance > 0.10 to indicate the absence of multicollinearity [65, 66]. These assessments ensure the robustness and reliability of the data, which is crucial for meaningful analysis and interpretation.

**Table 2:** Results of validity and reliability analysis.

Constructs Name	Number of Items	Cronbach Alpha	Collinearity Statistics	
			Tolerance	VIF
Effectiveness of Training on APA (ETAPA)	5	0.910	-	-
Reaction (RA)	4	0.939	0.173	5.775
Learning Skill (LS)	4	0.960	0.113	8.812
Behavior (BH)	4	0.965	0.149	6.733
Results (RE)	4	0.856	0.298	3.360

Note: ETAPA: Effectiveness of training on APA; RA: Reaction; LS: Learning skills; BH: Behavior; RE: Results (APA oriented performance)

Table 3 displays the descriptive statistics and the results of the correlation analysis for the variables. The average scores for the reaction and effectiveness of APA training were approximately 4.05. These findings indicate that, on average, the participants exhibited a higher level of drive to acquire knowledge and showed a favorable response to the APA training. The standard deviation measures the variability of data points from the mean, demonstrating the degree to which individual values deviate from the average, specifically with the behavior and effectiveness of training in comparison to other factors. The correlation matrix illustrates strong positive correlations among the variables like RA, LS, BH, RE, and ETAPA. All correlations are significant at the 0.01 level, indicating a robust relationship between these constructs. Specifically, reaction demonstrates the highest correlation with ETAPA, followed closely by learning skills and behavior, suggesting their influential roles in determining the effectiveness of APA training. These correlations indicate the influential roles these constructs play in shaping the overall effectiveness of the training. In essence, the data suggests that participants’ initial reactions, learning abilities, and behavioral changes are integral to the success of APA training programs, reinforcing the multidimensional nature of effective training interventions.

**Table 3:** Correlation matrix analysis.

Constructs	Mean	SD	RA	LS	BH	RE	ETAPA
Reaction (RA)	4.053	0.9476	1	.898^**^	.844^**^	.795^**^	.926^**^
Learning Skill (LS)	3.989	0.9823	.898^**^	1	.909^**^	.789^**^	.838^**^
Behavior (BH)	3.982	1.0371	.844^**^	.909^**^	1	.813^**^	.821^**^
Results (RE)	3.817	0.9373	.795^**^	.789^**^	.813^**^	1	.781^**^
Effectiveness of Training on APA (ETAPA)	4.005	0.8383	.926^**^	.838^**^	.821^**^	.781^**^	1

Note:

**. Correlation is significant at the 0.01 level (2-tailed).

## Evaluation using fsQCA

### Contrarian case analysis

Before applying fuzzy-set QCA, a contrarian case analysis in Table 4 was employed to effectively evaluate the number of cases within the collected sample that remained unaccounted for by the primary effects—these cases, which may not be considered in the outcomes of a traditional variance-based approach [61]. Scholars have underscored a common oversight in variable-level analyses, wherein researchers fail to acknowledge instances of association that contradict the main effect link [67]. Consequently, conducting a contrarian case analysis is advisable to identify potential favorable, unfavorable, or nonexistent connections within the dataset under examination [62].

**Table 4:** Results of contrarian case analysis.

			Effectiveness of post-training on APA								Effectiveness of post-training on APA				
			1	2	3	4	5				1	2	3	4	5
Reaction	(V = .573, Phi^2^ = 1.15, p < .001)	1	*11*	*2*	0	**0**	**0**	Behavior	(V = .458, Phi^2^ = .91, p < .001)	1	*9*	*4*	1	**0**	**0**
			*(84*.*6%)*	*(11*.*1%)*	(0.0%)	**(0.0%)**	**(0.0%)**				*(69*.*2%)*	*(22*.*2%)*	(11.1%)	**(0.0%)**	**(0.0%)**
		2	*1*	*10*	1	**2**	**0**			2	*3*	*4*	2	**3**	**0**
			*(7*.*7%)*	*(55*.*6%)*	(11.1%)	**(9.5%)**	**(0.0%)**				*(23*.*1%)*	*(22*.*2%)*	(22.2%)	**(14.3%)**	**(0.0%)**
		3	0	4	4	2	0			3	1	7	2	5	0
			(0.0%)	(22.2%)	(44.4%)	(9.5%)	(0.0%)				(7.7%)	(38.9%)	(22.2%)	(23.8%)	(0.0%)
		4	**1**	**2**	4	*11*	*4*			4	**0**	**1**	0	*8*	*2*
			**(7.7%)**	**(11.1%)**	(44.4%)	*(52*.*4%)*	*(40*.*0%)*				**(0.0%)**	**(5.6%)**	(0.0%)	*(38*.*1%)*	*(20*.*0%)*
		5	**0**	**0**	0	*6*	*6*			5	**0**	**2**	4	*5*	*8*
			**(0.0%)**	**(0.0%)**	(0.0%)	*(28*.*6%)*	*(60*.*0%)*				**(0.0%)**	**(11.1%)**	(44.4%)	*(23*.*8%)*	*(80*.*0%)*
Learning Skill	(V = .462, Phi^2^ = .92, p < .001)	1	*8*	*4*	1	**0**	**0**	Results	(V = .404, Phi^2^ = .80, p < .001)	1	*9*	*2*	0	***2***	***0***
			*(61*.*5%)*	*(22*.*2%)*	(11.1%)	**(0.0%)**	**(0.0%)**				*(69*.*2%)*	*(11*.*1%)*	(0.0%)	***(9*.*5%)***	***(0*.*0%)***
		2	*2*	*5*	3	**1**	**0**			2	*3*	*7*	1	**2**	**1**
			*(15*.*4%)*	*(27*.*8%)*	(33.3%)	**(4.8%)**	**(0.0%)**				*(23*.*1%)*	*(38*.*9%)*	(11.1%)	**(9.5%)**	**(10.0%)**
		3	2	8	1	8	0			3	1	5	4	5	3
			(15.4%)	(44.4%)	(11.1%)	(38.1%)	(0.0%)				(7.7%)	(27.8%)	(44.4%)	(23.8%)	(30.0%)
		4	**1**	**1**	3	*5*	*2*			4	**0**	**3**	1	*6*	*1*
			**(7.7%)**	**(5.6%)**	(33.3%)	*(23*.*8%)*	*(20*.*0%)*				**(0.0%)**	**(16.7%)**	(11.1%)	*(28*.*6%)*	*(10*.*0%)*
		5	**0**	**0**	1	*7*	*8*			5	**0**	**1**	3	*6*	*5*
			**(0.0%)**	**(0.0%)**	(11.1%)	*(33*.*3%)*	*(80*.*0%)*				**(0.0%)**	**(5.6%)**	(33.3%)	*(28*.*6%)*	*(50*.*0%)*

Notes

**Bolded** cases indicate contrarian examples, while cases in **italics** denote the primary effect. Contrarian case sets contrast with the main effect size (phi^2); ETAPA refers to the effectiveness of post-training on APA; RA stands for Reaction; LS for Learning Skills; BH for Behavior; RE signifies Results (APA-oriented performance).

To investigate associations, the research sample was divided using a quintile-based method in accordance with the recommendations of Pappas and Woodside [68] on contrarian case analysis. Cross-contingency analysis was then applied to test the quintiles. The outcome of the cross-contingency analysis between any two variables is shown as a 5 × 5 matrix, representing every possible configuration of the two variables across each quantile within the sample. Cases in the upper left and lower right corners signify primary effects, whereas those in the lower left and upper right corners are deemed contrarian since the main effects cannot account for them. The presence of cases at these corners indicates the existence of contrarian cases in the sample. Table 4 displays data on the cross-contingency of reaction, learning skills, behavior, results, and the effectiveness of the APA training. Together, these tables spotlight the contrarian cases within the sample, prompting the adoption of fsQCA to include counterfactual scenarios in examining the advanced training effectiveness of APA.

### Data calibration

In the context of fsQCA analysis, the outcome pertains to the effectiveness of the APA training, and each condition (reaction, learning skill, behavior, and results) is treated as a distinct set. For every case in these sets, a member score is assigned. Hence, data calibration involves assigning set member values to individual cases [69]. When multiple items measure a variable, a single value for each case is assigned by calculating the average of all items [68]. Following the approach outlined by Calabuig Moreno, Prado-Gascó [70], the calibration standards for each variable are set at the 0.05th percentile for full non-membership (fully out), 0.5th percentile for intersection (crossover point), and 0.95th percentile for full membership (full-in). Lastly, a logistic function was used to calibrate all the data to ensure they fulfilled the three requirements [28]. Table 5 shows how the fsQCA algorithm was used to skillfully execute the conversion of variables into calibrated sets.

**Table 5:** Compute thresholds using percentiles.

		ETAPA	Reaction	Learning Skill	Behavior	Results
N	Valid	71	71	71	71	71
	Missing	0	0	0	0	0
Percentiles	5 (Full out)	1.920	1.700	1.900	1.650	1.550
	50 (Crossover)	4.200	4.250	4.000	4.000	4.000
	95 (Full in)	5.000	5.000	5.000	5.000	5.000

In this study, the calibration method detailed below was employed to convert the scale into continuous fuzzy sets, explicitly utilizing the direct method in the fsQCA software version 3. The calibration function, Calibrate(x, n_1_, n_2_, n_3_), was applied, where ’x’ represents the research construct transforming, ’n_1_’ signifies full membership, ’n_2_’ denotes the crossover point, and ’n_3_’ indicates full non-membership. The algorithmic equation for these constructs is presented as follows:

Compute: ETAPA = calibrate (EffectivenessTrainingAPA, 5, 4.2, 1.92)

Compute: RA = calibrate (Reaction, 5, 4.25, 1.70)

Compute: LS = calibrate (LearningSkills, 5, 4, 1.90)

Compute: BH = calibrate (Behavior, 5, 4, 1.65)

Compute: RE = calibrate (Results, 5, 4, 1.55)

### Necessary condition analysis

Firstly, the importance of each condition must be assessed separately before we begin the examination of conditional configuration [69]. In this regard, a screening is carried out for both the condition’s presence and absence to determine whether any causal conditions are necessary for the effectiveness of the APA training program (Details in Table 6). As per Ragin [71], the concept of necessity indicates that a condition functions as a "superset of the result". This implies that, for every case in the data given, the fuzzy-set membership score of the outcome is lower than that of the causal conditions, as highlighted by Pappas, Mikalef [28]. In the same way, Schneider and Wagemann [72] argued that a condition must always have a consistency greater than 0.9 to be considered necessary. Ragin [71] argued that consistency is the ratio of instances in a sample that indicate a causal configuration leading to the desired outcome. The necessity implications are examined using the specialized software in fsQCA version 3.0, which generates the coverage and consistency results for each causal condition together with their negated values. As all the consistencies fall within the range of 0.5 to 0.9, we may proceed with our data calibration study. Table 6 shows the necessary condition results of high and low effectiveness of training on APA, in which the consistency of all conditions. It can be inferred that no necessary condition/factors influenced the high and low effectiveness of APA training. Below, we present a summary of the essential prerequisites:

**Table 6:** Analysis of necessary conditions for research constructs.

Condition Variables	Outcome variable: ETAPA		Outcome variable: ∼ ETAPA	
Conditions tested	Consistency	Coverage	Consistency	Coverage
RA	0.714285	0.837680	0.726968	0.731206
∼RA	0.770801	0.766988	0.838621	0.715699
LS	0.897697	0.828744	0.616229	0.487923
∼LS	0.445317	0.575000	0.783710	0.867905
BH	0.906332	0.819882	0.622026	0.482604
∼BH	0.428048	0.569044	0.767846	0.875478
RE	0.833333	0.847751	0.636059	0.554964
∼RE	0.562533	0.643135	0.825503	0.809452

Note: (∼) means the absence of. For instance: ∼ Learning skills = absence of high learning skills

### Generating the truth table

The fsQCA software created a truth table using the recalibrated data during this stage. This truth table is designed to contain 2^p rows, with ’p’ being the total number of conditions. Each row showcases every possible permutation of the conditions. For instance, in the scenario of having three conditions, the truth table will display 8 unique logical combinations among them. Following the creation of the truth table, frequency and consistency thresholds are set. The whole truth table can be found in the S2 Table. To ensure a minimal amount of empirical observations, a frequency cutoff threshold must be set. For more minor to medium samples, like less than 150 cases, a frequency cutoff point of 1 is applied. However, for larger-scale samples exceeding 150 cases, the cutoff point must be set at a value greater than 1, as stipulated by Zschoch [73]. Given that the study comprises 71 cases, a frequency cutoff point of "1" is instituted. It is important to note that employing a low consistency threshold can introduce errors and potentially allow false positive conditions [74]. Therefore, a relatively stringent consistency criterion, precisely > 0.80, is set. This criterion aligns with the established standard for fuzzy-set analysis. Table 7 contains the ‘set-theoretic consistency values’ for each configuration and the aggregate result, all surpassing the specified threshold (>0.8), as indicated by Pappas, Mikalef [28]. The consistency values gauge the accuracy of the approximation for each subset connection. At the same time, coverage assesses the empirical significance of a reliable subset and is based on the framework presented by Rihoux and Ragin [75] and also mentioned by Zschoch [73].

**Table 7:** Truth table after logical minimization.

RA	LS	BH	RE	Number	ETAPA	Cases	Raw consist.	PRI consist.	SYM consist
1	1	1	1	2	1	68, 71	0.959905	0.882558	0.892941
1	1	1	0	1	1	47	0.953887	0.813852	0.813853
0	1	1	1	14	1	1, 4, 5, 10, 12, 13, 21, 28, 29, 31, 36, 49, 52, 54	0.941418	0.850334	0.864341
0	1	1	0	2	1	14, 50	0.936192	0.774907	0.774908
0	0	0	1	2	1	6, 58	0.888039	0.363636	0.366534
0	1	0	0	2	1	23, 24	0.883333	0.268182	0.269406
1	0	0	0	1	0	67	0.743827	0.041571	0.041861

### Configurations for high and low effectiveness on APA training

In logic-based analysis, there are three solution sets: complex, parsimonious, and intermediate. Each solution set has a different approach to determining if a given combination is a part of the minimization process. Complex solutions include all possible combinations for analysis, while parsimonious solutions minimize them to the fewest viable choices. Intermediate solutions strike a balance by excluding combinations conflicting with theoretical understanding but retaining a cohesive number of answers to mitigate complexity. In interpreting fsQCA results, the recommended intermediate solutions, as Ragin [57] advocated, were adopted as the final solution sets. Put another way, sufficient solutions within the fsQCA framework independently lead to the desired outcome without the necessary conditions. The fsQCA analysis revealed three potential configurations of causal conditions that resulted or contributed to training effectiveness. The findings of the fsQCA model are presented in Table 8:

**Table 8:** Configurational paths of post-training effectiveness on APA.

No.	Path of Solutions	Raw Coverage	Unique Coverage	Consistency
A	Model: ETAPA = ƒ (RA*LS*BH*RE)	Frequency cutoff: 1; Consistency cutoff: 0.883333		
1	Learning*Behavior	0.872318	0.362637	0.875525
2	∼Reaction*Learning*∼Results	0.482993	0.005756	0.908912
3	∼Reaction*∼Learning*∼Behavior*Results	0.334118	0.029304	0.888039
	Solution consistency	0.853314		
	Solution coverage	0.916274		
B	Model: ∼ETAPA = ƒ (RA*LS*BH*RE)	Frequency cutoff: 1; Consistency cutoff: 0.934631		
4	∼Learning*∼Behavior*∼Results	0.644906	0.089688	0.950113
5	∼Reaction*∼Behavior*∼Results	0.616230	0.061012	0.958254
6	∼Reaction*∼Learning*∼Behavior	0.601587	0.046369	0.935928
	Solution consistency		0.910635	
	Solution coverage		0.752288	

Note: ETAPA: Effectiveness of training on APA; RA: Reaction; LS: Learning skill; BH: Behavior; RE: Results (APA oriented performance)

Table 9 provides a summary of configurations with solutions: black circles (●) signify the presence of a condition, crossed-out circles (⊗) indicate its absence, and dashed circles (ꟷ) signify a "don’t care" situation where the outcome does not depend on whether these causal conditions are present or absent, as explained by Mikalef and Krogstie [76]. The fsQCA analysis reveals three distinct paths leading to the perceived effectiveness of APA training.

**Table 9:** Configurations leading to high and low effectiveness on APA training.

	Configurational models with solutions					
Path Functions	ETAPA = ƒ (RA*LS*BH*RE)			∼ETAPA = ƒ (RA*LS*BH*RE)		
Condition Variables	1	2	3	4	5	6
Reaction (RA)	ꟷ	⊗	⊗	ꟷ	⊗	⊗
Learning Skill (LS)	●	●	⊗	⊗	ꟷ	⊗
Behavior (BH)	●	ꟷ	⊗	⊗	⊗	⊗
Result (RE)	ꟷ	⊗	●	⊗	⊗	ꟷ
Consistency	0.875525	0.908912	0.888039	0.950113	0.958254	0.935928
Raw Coverage	0.872318	0.482993	0.334118	0.644906	0.616230	0.601587
Unique Coverage	0.362637	0.005756	0.029304	0.089688	0.061012	0.046369
Overall Solution Consistency	0.853314			0.910635		
Overall Solution Coverage	0.916274			0.752288		

Note: Black circles (●) indicate the presence of a condition, circles with a cross-cut (⊗) indicate its absence or negation of a condition, and Blank spaces (ꟷ) indicate the condition may not be either present or absent.

For Model A, none of the solutions alone can achieve a high level of effectiveness in APA training. Specifically, Solution 1 shows (Learning Skill*Behavior) that learning skills and behavior collectively influence post-training effectiveness through distinct paths. This implies that the joint presence of these factors significantly affects the perceived effectiveness (consistency = 0.875525) and coverage = 0.872318) of the APA training program. This could be understood as the existence of learning skills and behaviors, which are likely to predict a high positive relation to training effectiveness. Solution 2’s configuration (∼Reaction*Learning*∼Results) implies that a low trainee’s reaction and result (outcome on APA) are sufficient to improve APA training effectiveness, even though learning skill is prevalent. It means that even if trainee reactions are suboptimal and results on the APA are low impact, a high learning skill level (consistency = 0.908912 and coverage = 0.482993) can still enhance the training program’s effectiveness. In the analysis, Solution 3 indicates that configuration (∼Reaction*∼Learning*∼Behavior*Results) signifies that low levels of trainee reaction, learning skill, and behavior, despite dominant results, are sufficient to enhance the effectiveness of APA training. This implies that, even when trainee reactions, learning skills, and behavior are not quite optimal, the training program’s effectiveness can be elevated (consistency = 0.888039 and coverage = 0.3341180), particularly in the presence of dominant results on APA.

Alternatively, In Model B, the factors that hinder the effectiveness of APA training—but three different configurations were identified, which do not mirror the configurations that promote the APA training effectiveness. The configuration (∼Learning*∼Behavior*∼Results) suggests that the absence of learning skills, behavior, and favorable results collectively enhances APA training effectiveness (consistency = 0.950113 and coverage = 0.644906). This configuration highlights the significance of these three factors in influencing the perceived impact of APA training, underscoring their absence as a contributing factor to enhanced effectiveness. Also, configuration (∼Reaction*∼Behavior*∼Result) signifies that when trainee reaction, behavior, and favorable results on APA are absent, the training program’s overall effectiveness is enhanced. This configuration emphasizes the collective impact of the absence of these factors as a critical contributor to the heightened effectiveness of APA training (consistency = 0.958254 and coverage = 0.61623). In fine, configuration (∼Reaction*∼Learning*∼Behavior) in the fsQCA context implies that when there are no discernible trainee reactions, learning skills, and behaviors in the context of APA training, there is a distinct enhancement in the overall effectiveness of the training program. The absence of these three elements together highlights their substantial contribution to the increased effectiveness of APA training (consistency = 0.935928 and coverage = 0.601587).

Table 9 highlights that three causal configurations hold empirical significance in both models. When comparing Model A with Model B, Model A shows a slightly lower level of consistency at 0.853314, but it reaches a better level of coverage at 0.916274. In contrast, Model B demonstrates a better level of consistency at 0.910635; however, it has a lesser coverage of 0.752288. The configurations represent amalgamations of relevant circumstances, and the absence of any single condition is adequate to explain post-training effectiveness. Following scholarly discourse, coverage ratings serve as a means to assess the practical significance of a set of causal factors. Empirical relevance pertains to the degree to which a specific causal condition can elucidate an observable outcome. Drawing on the arguments mentioned above and pragmatic solutions, Table 10 presents the two topologies of trainees’ post-training evaluation and their related pathways to the training program. These topologies are trainees’ learning-behavior-results and reaction-learning-behavior-result. It offers a clear and precise consideration appropriate for an academic setting, focusing on the structural factors that impact the effectiveness of the training program.

**Table 10:** Topological framework of APA training effectiveness.

Label/Identification	Clarification according to paths	Key insights
Learning-behavior-result (A1 and A4)	1. High learning skills with behavior 2. Collectively, this contributes to low learning skills and behavior, along with favorable results.	1. Integrating advanced learning skills and positive behaviors improves the success and effectiveness of post-training results. 2. The concrete results of the APA training may offset deficiencies in learning abilities and behaviors, emphasizing the importance of quantifiable outcomes in post-training evaluations.
Reaction-learning-behavior-result (A2, A3, A5 and A6)	1. High learning skills along with better reaction and result 2. Favorable result with low reaction, skills and behavior 3. Suboptimal reaction, behavior and results 4. Low impact of reaction, skill and behavior	1. The comprehensive and favorable alignment of these parameters greatly enhances the effectiveness of the training. It implies a holistic engagement and effective utilization of acquired knowledge. 2. The final success of the APA training program may not exclusively hinge on instant favorable responses or exemplary skills and behavior. It emphasizes the significance of quantifiable positive outcomes as a crucial factor in assessing the effectiveness of the training program, even if other factors may be less prominent. 3. Even when participants exhibit less-than-ideal reactions, behavior, and results, other factors, such as the learning content or delivery methods, might be at play that contribute to a level of success or positive outcomes. 4. When trainees exhibit minimal impact regarding reactions, learning skills, and behaviors, there might be challenges in achieving desired outcomes. It underscores the importance of addressing these aspects during the training design and delivery phases to enhance overall effectiveness.

**Fig 2:**
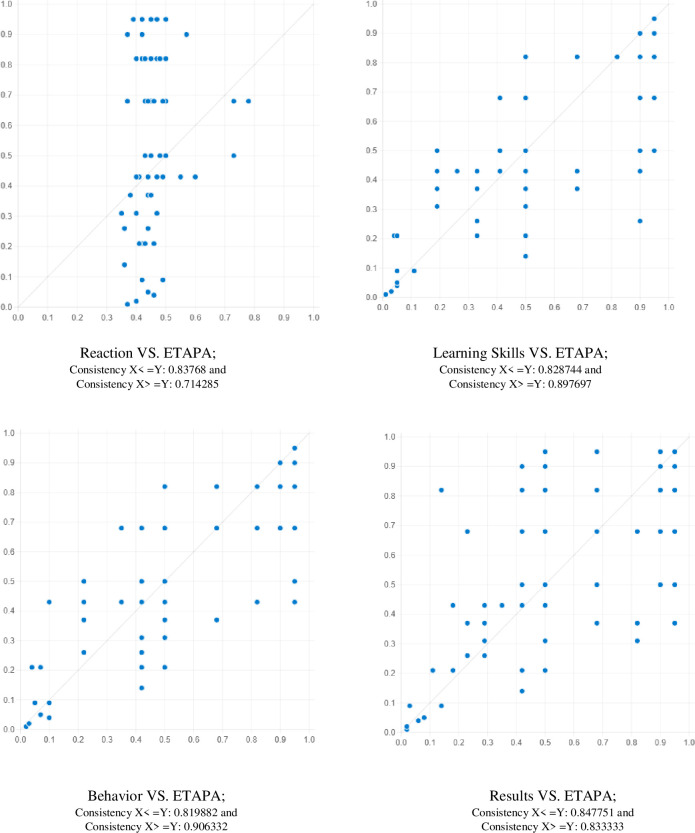
XY Plot of post-training effectiveness on APA.

Fig 2 displays XY plots illustrating the correlation between the outcome and the related variables. This enables an analysis of the instances where particular variables exhibit higher or diminished values, are not present, or display no apparent correlation that leads to the outcome. This research is carried out over many scenarios with unique independent factors.

### Artificial neural network (ANN) analysis

**Fig 3:**
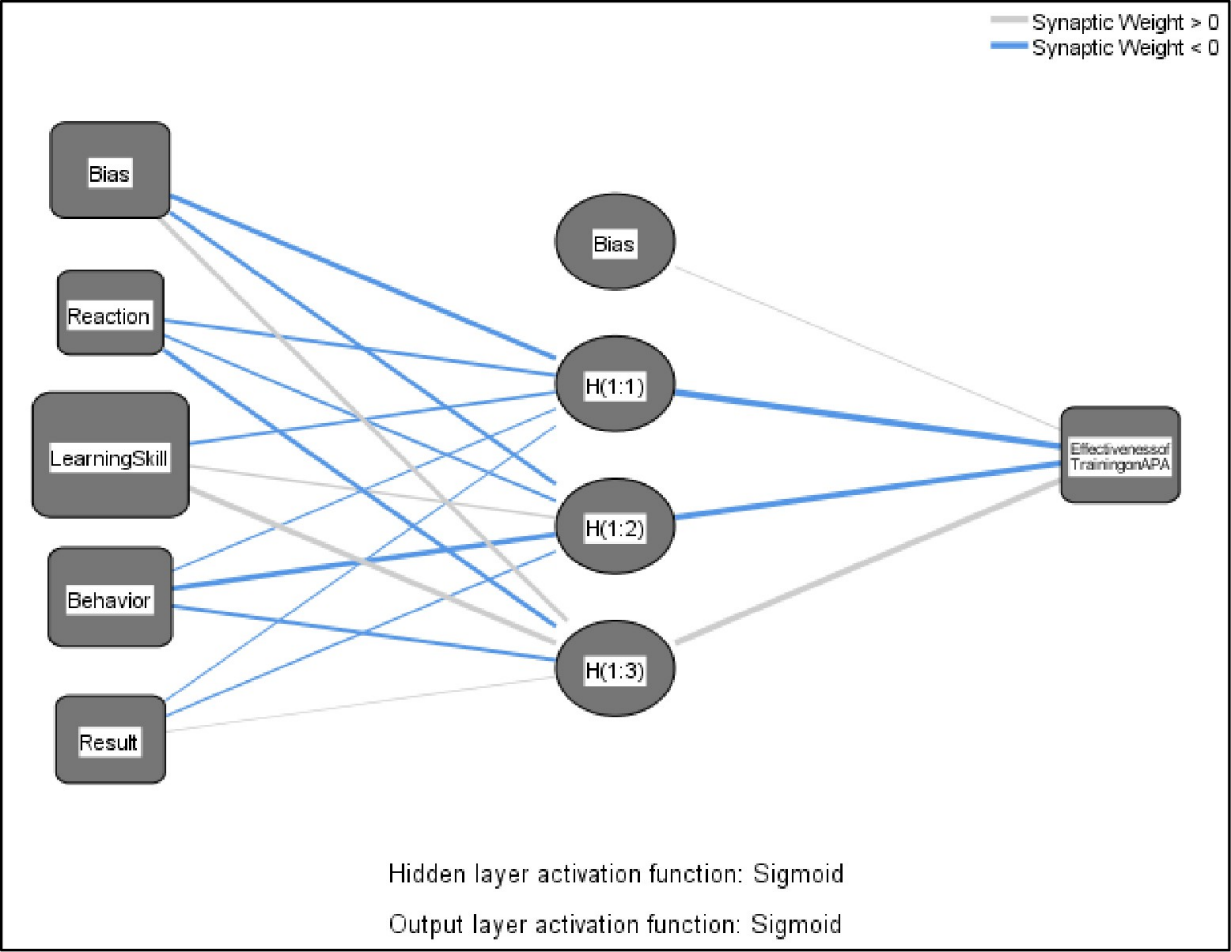
Visual representation of the ANN structure.

**Fig 4:**
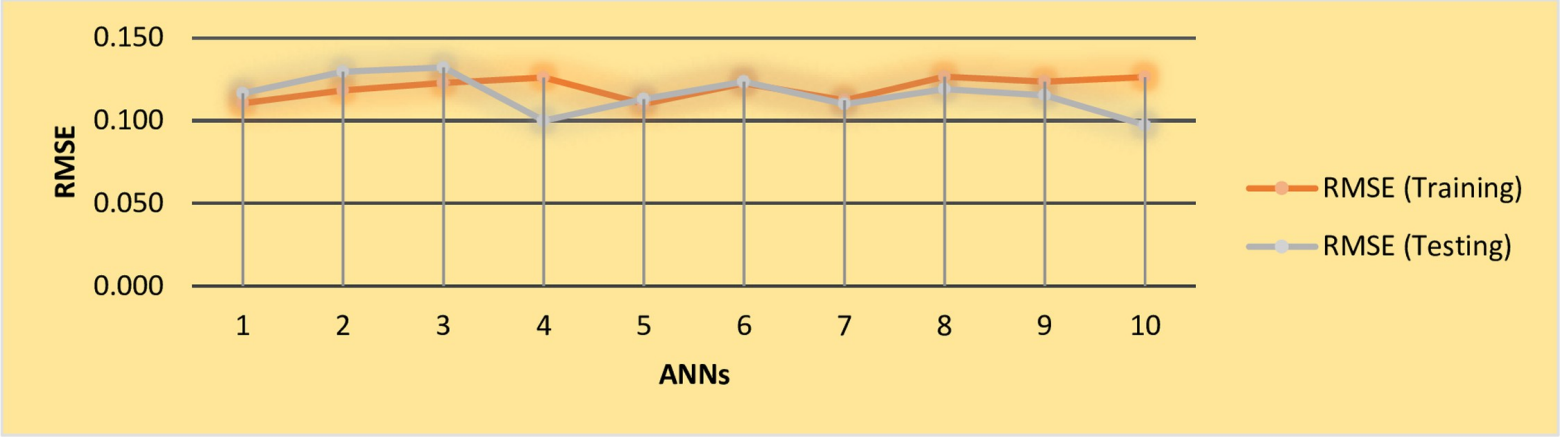
RMSE values (training and test data).

The study conducted additional analysis in light of potential non-linear relationships between the independent and dependent variables. This study utilized ANN to examine the normalizing importance of influential factors concerning the outcome variable [77, 78]. Additionally, the prediction outputs obtained from the ANN display greater precision than those obtained from the fsQCA approach. Hence, employing an integrated fsQCA-ANN technique provides a more comprehensive explanation of the prediction model, thereby improving the robustness of final results. ANN analysis was performed using the Multilayer Perceptron (MLP) algorithm in SPSS version 26. The present work employs an MLP feed-forward back propagation multilayer training approach with a sigmoid activation. The research study applied a ten-fold cross-validation procedure, which involved dividing the data into separate training and test sets to reduce the risk of overfitting, as presented in Table 11. More precisely, the dataset was partitioned, with 70% of the data allocated for training and the remaining 30% for testing, following the methodology described by Liébana-Cabanillas, Marinković [79]. The study confirms the findings of the ANN by evaluating the accuracy using the root mean square error [79, 80] and verifies the findings of the ANN, and the researchers utilized a performance metric known as RMSE [81]. The present study used an ANN approach to analyze the relationship between reaction (RA), learning skill (LS), behavior (BH), and results (RE). These variables were operationalized as the input layer (also known as neurons) in the ANN framework. The effectiveness of the training on APA (ETAPA) was considered the model’s outcome layer, as shown in Fig 3. RMSE was calculated using the following equations: Eqs 1 and 2, where SSE represents the sum of squared error and MSE represents the mean squared prediction error. The average RMSE values range from 0.4 to 0.5 for both the training and testing sets, which are graphically depicted in Fig 4.


$$\text{MSE}=\left[\frac{1}{n}\right]x\;SSE$$ (1)

RMSE=√MSE (2)

**Table 11:** Sample size, SSE, and RMSE values during training and testing phases.

Input and Output Variables	Input: Reaction; Learning skill; Behavior; Results						
	Output: Effectiveness of training program on APA						
Neural Networks	Training			Testing			Total Samples
	SSE	RMSE	N	SSE	RMSE	N	
ANN1	0.612	0.1106	50	0.285	0.1165	21	71
ANN2	0.770	0.1183	55	0.269	0.1297	16	71
ANN3	0.783	0.1227	52	0.332	0.1322	19	71
ANN4	0.667	0.1260	42	0.29	0.1000	29	71
ANN5	0.655	0.1101	54	0.217	0.1130	17	71
ANN6	0.765	0.1225	51	0.305	0.1235	20	71
ANN7	0.593	0.1123	47	0.291	0.1101	24	71
ANN8	0.705	0.1266	44	0.382	0.1189	27	71
ANN9	0.793	0.1235	52	0.253	0.1154	19	71
ANN10	0.799	0.1264	50	0.199	0.0973	21	71
Mean	0.714	0.1199		0.2823	0.1157		
Standard deviation	0.074	0.0063		0.0503	0.0108		

The results affirm the reliability and accuracy of the ANN models, as the RMSE values are below 0.50, indicating robust relationships between predictors and outputs (Chong, 2013). Additionally, the RMSE scores for both the training and test data provide evidence that all predictor variables substantially affect ETAPA. Each value has a magnitude smaller than 0.50 and is close to one another, indicating high accuracy and reliability in the model fit.

### Sensitivity analysis

Sensitivity analysis is a technique that assesses the normalized significance of each input by comparing their relative importance and expressing this as a percentage. This research aims to identify how changes in independent variables affect the dependent variable, calculate the average significance of reaction, learning skills, behavior, and results as independent variables, and determine their effect on training effectiveness on APA. Table 12 shows the normalized relative importance of each variable in percentages. The data reveal that learning skill emerges as the most significant predictor of ETAPA, holding 100% normalized relative importance, followed by behavior, results, and reaction with 80%, 54%, and 30% normalized relative importance, respectively. The congruence of findings from both fsQCA and ANN analyses confirms that learning skill is the paramount variable, supporting the accuracy of the study’s predicted model. This agreement highlights the model’s reliability.

**Table 12:** Sensitivity analysis with normalized importance.

ANN Models	Reaction	Learning Skill	Behavior	Results
ANN1	0.3178	1.0000	0.5650	0.3701
ANN2	0.3698	1.0000	0.5916	0.5131
ANN3	0.0536	1.0000	0.9206	0.6221
ANN4	0.2846	1.0000	0.8678	0.8040
ANN5	0.6550	1.0000	0.4554	0.2826
ANN6	0.4254	0.9865	1.0000	0.8553
ANN7	0.4237	1.0000	0.6485	0.5900
ANN8	0.1655	0.8398	1.0000	0.3515
ANN9	0.1565	0.9430	1.0000	0.5162
ANN10	0.1096	1.0000	0.7807	0.3402
Mean importance	0.2961	0.9769	0.7830	0.5245
Normalized importance %	30.31%	100.00%	80.15%	53.69%
Ranking	4	1	2	3

## Comparison of fsQCA and ANN approach

Table 13, a comparison of fsQCA and ANN approaches in assessing APA training effectiveness, showcases distinct strengths: fsQCA is adept at uncovering complex causal relationships in smaller datasets, revealing various pathways to desired outcomes, whereas ANN excels in identifying patterns and making predictions across larger datasets by integrating both quantitative and qualitative way. The combined use of fsQCA and ANN in evaluating APA training effectiveness through Kirkpatrick’s four-level model highlights that engaging training sessions boost trainee satisfaction, tailored content improves learning, practical application and support encourage behavioral change, and strategic alignment and management backing are essential for achieving significant organizational benefits. This comprehensive analysis underscores the importance of tailored training approaches and organizational support in achieving effective APA training outcomes.

**Table 13:** Results comparing the fsQCA and ANN approach.

Path connection based on Model	fsQCA	ANN	Remarks
Model: ETAPA = ƒ(RA*LS*BH*RE) Learning*Behavior ∼Reaction*Learning*∼Results ∼Reaction*∼Learning*∼Behavior*Results	Each of these configurations provides insight into the subtle factors that impact the effectiveness of APA training.	Importance of LS is the most influential variable, with a normalized relative value of 100%, followed by BH with 80% importance, RE with 54% importance, and RA with 30% importance.	The consistency between the fsQCA and ANN findings is evidence that the model can predict the effectiveness of post-APA training accurately.
Model: ∼ETAPA = ƒ(RA*LS*BH*RE) ∼Skill*∼Behavior*∼Results ∼Reaction*∼Behavior*∼Results ∼Reaction*∼Skill*∼Behavior	This means that the presence of any of these conditions is enough to produce the outcome, and the absence of any of these conditions will prevent the outcome from occurring.	These configurations collectively indicate conditions that result in a negative effect on the effectiveness of post-APA training.	The identified configurations show that any one condition is enough to produce the outcome. In contrast, lacking these elements indicates that APA training in the ANN technique is ineffective.

## Discussion

Trainees are more inclined to attain elevated levels of training when the organization implements one of the four levels of training evaluation criteria delineated by Kirkpatrick’s model, particularly those featuring complete training programs on APA. Conversely, the trainees’ lacking of performance can be attributed to four distinct training program designs on APA. To optimize the effectiveness of the APA training, it is necessary to revamp training programs to conform to established principles for success. In essence, there are multiple avenues for attaining favorable training and evaluation of training programs, which can motivate training coordinators or organizers. The research employed the fsQCA, and ANN approaches within the outlined evaluation model to discern diverse configurations that would lead to enhanced effectiveness of APA training within the public sector. Antecedent conditions considered in the study encompassed reaction (RA), learning (LS), behavior (BH), and results (RE). Upon evaluating the necessary conditions (refer to Table 6), the findings indicate that none of the conditions is consistently useful. Consequently, the results indicate that a combination of conditions is conducive to enhancing APA effectiveness in public sector training.

In examining Model A, considering the three configurations mentioned in Table 8, a detailed discussion unfolds regarding the implications for training effectiveness in the context of APA training. Configuration 1 suggests that the combination of high learning skills and positive behavior is associated with a positive outcome in ETAPA, providing support for the modeling of ETAPA [50]. This underscores the importance of not only acquiring knowledge of related APA training but also proving positive behavioral attributes for optimal training effectiveness. On the other hand, Configuration 2 implies that low reaction, high learning skills, and an absence of favorable results collectively contribute to APA training effectiveness. In this sense, this goes against the grain of common wisdom, highlighting the necessity of closely examining responses and outcomes in addition to learning abilities. In essence, it encourages a re-evaluation of the factors deemed critical for training success, emphasizing the importance of not only participants’ immediate reactions but also their aptitude for learning skills and the final results attained. This insight confirms [46] the necessity of adopting a more in-depth and subtle view while evaluating the influence of training programs like APA. Likewise, Configuration 3 posits that low reaction, low learning skill, and low behavior, despite positive results, are sufficient for enhancing APA effectiveness. This highlights the complex interplay of factors and implies that desired outcomes can be achieved even in the absence of certain conventional or familiar elements. Overall, these configurations shed light on the multifaceted nature of training effectiveness in the context of APA, highlighting the need for a holistic approach that takes into account other variables beyond conventional beliefs [82]. The findings pave the way for a more detailed comprehension of the intricacies involved in the post-training evaluation of APA. This offers valuable insights for training program design and execution of training program in Model A.

In addition, looking at three configurations within Model B reveals interesting findings about how APA is evaluated after training. Configuration 4 indicates that despite initial negative reactions, positive learning skills, behavior, and results contribute to the effectiveness of the APA training. This underlines how crucial it is to concentrate on improving participants’ learning skills and behavior as well as attaining desired results—even in the absence of immediate positive reactions. Configuration 5 highlights the importance of addressing factors beyond initial reactions, focusing on behavior and outcomes. It clarifies that to ensure training success, it is important to consider variables other than participants’ initial reactions, such as their behavior and the final results attained. Configuration 6 displays the importance of refining learning skills and behavior to achieve good training outcomes, regardless of initial reactions. These findings question conventional perspectives on the effectiveness of training, highlighting the importance of a holistic strategy when constructing APA training programs [25]. The above discussion explores its practical implications for the public sector, emphasizing the significance of considering many aspects to achieve post-training evaluation.

In the realm of assessing the effectiveness of the APA training, this neural network model has identified four pivotal variables gleaned from the fsQCA that are integral to predicting the APA training effectiveness. The neural network model exhibits a commendable level of prediction accuracy, as already mentioned by the preceding scholars [83]. The analysis of the artificial neural network reveals varying levels of importance for distinct factors in predicting the effectiveness of the training program. *Learning skill*s emerge as the most influential factor, with a mean importance of 0.9769 and a normalized importance of 100%, ranking it highest among all variables. This highlights the critical role of participants’ learning skills in ascertaining training effectiveness. *Behavior* follows closely behind, with a mean importance of 0.7830 and normalized importance of 80.15%, highlighting its significant contribution to APA training outcomes. The *Results* are ranked third in terms of relevance, with a mean importance score of 0.5245 and a normalized importance value of 53.69%. Notably, although the *Reaction* has a lower mean value of 0.2961, it is still relevant in the prediction model, highlighting the multifaceted nature of training effectiveness. These findings point out that it is essential to prioritize the improvement of participants’ learning skills and behavior, as well as the attainment of intended outcomes, to maximize the effectiveness of post-training APA. Hence, it is hypothesized that the ANN analysis could enhance the fsQCA by assigning a numerical ranking to the conditions based on their relative significance to the desired results.

## Implications and contributions

### Policy implications

Based on the study using fsQCA and ANN, it is crucial to emphasize the significant role of learning skills and behavior in predicting the effectiveness of the APA training. In particular, the normalized importance rankings show that LS ranks highest, then BH, indicating that civil servants should prioritize initiatives aimed at improving participants’ learning skills and behavior to maximize the impact of APA training. Additionally, while reaction and result play significant roles in training effectiveness, their relative importance is lower than LS and BH. Therefore, bureaucratic policymakers should not overlook the importance of addressing participants’ initial reactions and achieving desirable outcomes; instead, they should focus more attention on fostering conducive learning environments and promoting positive behavior among trainees. Moreover, the particular configurations found in both models provide insightful information on the interactions between various elements affecting training effectiveness. For instance, Model A asserts that effective training outcomes can be linked to positive learning skills and behavior, even if there are no favorable initial reactions or results. In contrast, Model B emphasizes the significance of tackling adverse emotions, behavior, and results to enhance training effectiveness. Through the above approach, this study is intended to caution against the potential pitfalls associated with certain undesirable pathways. The hybrid technique enables such insights into the ramifications of different trajectories. The deliberate selection of this analytical approach allows bureaucratic policymakers and trainers to identify and outline alternate course of action, catering to situations that result in high and low levels of training effectiveness regarding APA. Ultimately, policymakers should take these findings into account when designing, implementing, and evaluating APA training programs. By prioritizing the development of learning skills and fostering positive behavior among participants, the effectiveness of this APA program can be greatly enhanced, which will contribute to improving performance and accountability within the public sector. Further, it is crucial to prioritize initiatives aimed at addressing negative reactions, behavior, and outcomes to mitigate potential obstacles to the success of the training.

### Methodological contributions

The combination of hybrid analysis with fsQCA and ANN in this study provides a systematic methodological contribution to the post-training evaluation literature. Firstly, the study introduces a novel way of combining fsQCA-ANN approaches to evaluate the effectiveness of the APA training program. This methodology allows for a holistic evaluation that leverages the strengths of both qualitative and quantitative methods. Secondly, employing QCA, the research examines various configurations of conditions, including trainee reactions, learning skills, behavior, and results, facilitating an extensive analysis that elucidates visible paths and combinations that contribute to training efficacy. Thirdly, applying ANN, the research evaluates the normalized importance rankings of several elements that impact post-training evaluation. This quantitative analysis provides insights into the relative importance of each factor, highlighting key drivers of training effectiveness, such as learning skills and behavior. Fourthly, by considering Kirkpatrick’s dimensions of training effectiveness, such as learner reactions, learning skills, behavior, and overall results, the study adopts a holistic perspective on training evaluation. This methodology offers a deeper insight into the intricate interrelationships inherent within training programs. Finally, the study translates its methodological insights into actionable policy implications for civil servants involved in designing and implementing APA training. These recommendations for enhancing training effectiveness in the public sector are derived from the collective results of fsQCA and ANN analyses. In summary, the study’s methodological contributions are centered around its inventive approach to evaluating training, a thorough analysis of training elements, and practical suggestions for policy creation and execution.

## Conclusions

In conclusion, this study provides valuable insights into the post-training evaluation of Annual Performance Agreement (APA) training in the public sector. By employing a novel approach that integrates qualitative comparative analysis and artificial neural network techniques, this research has contributed to a deeper understanding of the factors influencing APA training effectiveness. Our findings underscore the significance of learning skills and behavior as critical determinants of training outcomes. It became clear from the analysis of normalized importance rankings produced by ANN that these variables are crucial in determining how effective APA training is. This highlights the importance of focusing on enhancing participants’ learning skills and behavior to ensure successful training outcomes. Moreover, our study identified various configurations of conditions that contribute to improved training effectiveness. These configurations challenge conventional notions of training evaluation by emphasizing the need to consider multiple factors beyond participants’ initial reactions. By addressing factors such as behavior and the ultimate outcomes achieved, APA training can be redesigned to better align with desired objectives. The study’s novelty lies in its dual analytical approach to post-training evaluation, explicitly focusing on trainees’ evaluations. To summarize, this research adds to the current knowledge on evaluating training outcomes after completion and emphasizes the significance of using an accurate approach to measure the effectiveness of training. By integrating qualitative and quantitative techniques, this study offers valuable insights that can inform policy and practice in the field of public sector training and development.

## Limitations and directions for further research

This study presents several noteworthy limitations that merit consideration. Firstly, the sample size, encompassing only 71 in-service civil servants, while adequate for initial post-training evaluation, may impede the extrapolation of the findings to a wider civil servant population in Bangladesh, let alone to those in globally diverse contexts with varying training frameworks. The narrow sample scope potentially limits the universality and applicability of the research outcomes. Secondly, the study’s methodological approach, which integrates fsQCA with ANN, presents a sophisticated analytical framework. However, this complexity might pose substantial challenges in terms of replication and comprehensive understanding for other researchers or practitioners, particularly for those who are not versed in these advanced methods. Thirdly, the research heavily relies on subjective survey data. While insightful, such data is inherently prone to biases, personal perceptions, and potential misunderstandings by respondents. This reliance raises concerns about the extent to which these self-reported measures accurately reflect the actual effectiveness of the training in enhancing job performance or behavioral changes. Fourthly, the research’s scope primarily focuses on the immediate aftermath of the training, neglecting a long-term perspective. An evaluation of the enduring impact of the training on behavior and results is essential to fully comprehend its long-standing effectiveness, a facet not addressed in this study. Fifthly, there appears to be an omission in considering external variables that could significantly influence the training’s effectiveness. Factors such as shifts in organizational policies, fluctuating economic conditions, or the prevailing political landscape could have substantial repercussions on how trainees assimilate and apply the acquired skills. In synthesizing these limitations, it is crucial to recognize that their acknowledgement serves not to diminish the study’s value but rather to provide a more rounded and critical understanding of its scope and applicability.

Consequently, we extend an invitation to the academic community to replicate and extend this study in varied contexts. For future research endeavors, it is recommended to broaden the scope and heterogeneity of participant cohorts, encompassing a more extensive array of public employees. This strategy is vital for augmenting the universality and applicability of the research outcomes. A longitudinal approach is imperative for a comprehensive understanding of the enduring effects of training, transcending the confines of immediate post-training assessments. Furthermore, executing comparative studies within the unique context of Bangladesh and in a global arena could yield insightful revelations into the efficacy of diverse training paradigms. Integrating qualitative methodologies, such as in-depth interviews and Focus Group Discussions, would facilitate a richer and more intricate understanding of the trainees’ subjective experiences and perceptions. It is also prudent to consider cross-disciplinary methodologies, drawing from psychology, sociology, and organizational behavior, to provide a more enriched and multifaceted analytical lens. Lastly, the adoption of avant-garde analytical techniques, such as machine learning, holds the promise of surmounting certain inherent limitations of conventional statistical methods, thereby ushering in novel analytical perspectives. These suggested research trajectories are collectively aimed at deepening the comprehension and refining the assessment of training effectiveness within the domain of public administration.

## Supporting information

S1 Table Measurement items with sources. (DOCX)

S2 Table Truth table. (DOCX)

S1 Data (CSV)

S1 Appendix (DOCX)
