# Supplementary material for: Enhancing post-training evaluation of annual performance agreement training: A fusion of fsQCA and artificial neural network approach
Source: PLoS One. 2024 Jun 25;19(6):e0305916. doi: 10.1371/journal.pone.0305916 (PMC11198856; doi:10.1371/journal.pone.0305916)
Supplement: S1 Table — (DOCX) [file pone.0305916.s001.docx]

**Table SI** Measurement items with sources.

| **SI** | **Constructs** | **Scale Items** | **Code** |
| --- | --- | --- | --- |
| A. | Reaction | Annual performance agreement (APA) is a powerful instrument for promoting accountability. | RA1 |
|  |  | The training materials for the APA were captivating and thought provoking. | RA2 |
|  |  | The format of the APA training courses was meticulously organized and easy to follow. | RA3 |
|  |  | The APA training fulfilled my expectations and was directly applicable to my career. | RA4 |
| B. | Learning | The APA training enhanced my learning of the topics related to the annual performance agreement. | LS1 |
|  |  | The APA training has equipped me with additional knowledge and abilities directly applicable to my job responsibilities. | LS2 |
|  |  | The training materials successfully communicated the fundamental principles of the annual performance agreement. | LS3 |
|  |  | I received knowledge about the APA format, including its processes, measurement, and focal point function. | LS4 |
| C. | Behavior | I have implemented the knowledge and skills acquired from the APA training in my professional duties within the organization. | BH1 |
|  |  | The APA training has positively influenced my work behaviour and practices. | BH2 |
|  |  | I have gained more confidence in carrying out activities linked to the annual performance agreement after the course. | BH3 |
|  |  | I possess knowledge of APA guidelines and understand my responsibilities following the completion of training. | BH4 |
| D. | Results | The APA training has contributed to improved overall performance in my role. | RE1 |
|  |  | The abilities acquired from the APA training have had a favorable influence on the results achieved by the organization. | RE2 |
|  |  | The APA training has been effective in achieving its intended goals. | RE3 |
|  |  | The APA is in accordance with the organization’s vision and objectives. | RE4 |
| E. | Effectiveness on APA Training | The APA training substantially enhanced my understanding of performance management ideas. | EAPA1 |
|  |  | The training materials effectively conveyed the essential elements of the annual performance agreement. | EAPA2 |
|  |  | The APA training positively influenced my ability to set and achieve performance goals. | EAPA3 |
|  |  | I have observed the positive impact on the organization’s overall results due to the APA training. | EAPA4 |
|  |  | I believe the APA training has prepared me well for handling performance-related responsibilities. | EAPA5 |
