# Supplementary material for: Enhancing post-training evaluation of annual performance agreement training: A fusion of fsQCA and artificial neural network approach
Source: PLoS One. 2024 Jun 25;19(6):e0305916. doi: 10.1371/journal.pone.0305916 (PMC11198856; doi:10.1371/journal.pone.0305916)
Supplement: S2 Table — (DOCX) [file pone.0305916.s002.docx]

**Table S2** Truth table

| **RA** | **LS** | **BH** | **RE** | **Number** | **Raw consist.** | **PRI consist.** | **SYM consist.** |
| --- | --- | --- | --- | --- | --- | --- | --- |
| 0 | 0 | 0 | 0 | 14 | 0.665059 | 0.067164 | 0.067264 |
| 0 | 1 | 1 | 1 | 14 | 0.941418 | 0.850334 | 0.864341 |
| 0 | 1 | 0 | 0 | 2 | 0.883333 | 0.268182 | 0.269406 |
| 0 | 1 | 1 | 0 | 2 | 0.936192 | 0.774907 | 0.774908 |
| 0 | 0 | 0 | 1 | 2 | 0.888039 | 0.363636 | 0.366534 |
| 1 | 1 | 1 | 1 | 2 | 0.959905 | 0.882558 | 0.892941 |
| 1 | 0 | 0 | 0 | 1 | 0.743827 | 0.041571 | 0.041861 |
| 1 | 1 | 1 | 0 | 1 | 0.953887 | 0.813852 | 0.813853 |
| 1 | 1 | 0 | 0 | 0 | - | - | - |
| 0 | 0 | 1 | 0 | 0 | - | - | - |
| 1 | 0 | 1 | 0 | 0 | - | - | - |
| 1 | 0 | 0 | 1 | 0 | - | - | - |
| 0 | 1 | 0 | 1 | 0 | - | - | - |
| 1 | 1 | 0 | 1 | 0 | - | - | - |
| 0 | 0 | 1 | 1 | 0 | - | - | - |
| 1 | 0 | 1 | 1 | 0 | - | - | - |
