# Supplementary material for: Enhancing post-training evaluation of annual performance agreement training: A fusion of fsQCA and artificial neural network approach
Source: PLoS One. 2024 Jun 25;19(6):e0305916. doi: 10.1371/journal.pone.0305916 (PMC11198856; doi:10.1371/journal.pone.0305916)
Supplement: S1 Appendix — (DOCX) [file pone.0305916.s004.docx]

**Appendix A**

Table 8 Measurement items, with sources

| **SI** | **Constructs** | **Scale Items** | **Code** | **Sources** |
| --- | --- | --- | --- | --- |
| A. | Reaction | Annual performance agreement (APA) is a powerful instrument for promoting accountability. | RA1 | (Hsu & Chen, 2021) |
|  |  | The training materials for the APA were captivating and thought-provoking. | RA2 |  |
|  |  | The format of the APA training courses was meticulously organized and easy to follow. | RA3 |  |
|  |  | The APA training fulfilled my expectations and was directly applicable to my career. | RA4 |  |
| B. | Learning | The APA training enhanced my learning of the topics related to the annual performance agreement. | LS1 | (Alsalamah & Callinan, 2021) |
|  |  | The APA training has equipped me with additional knowledge and abilities directly applicable to my job responsibilities. | LS2 |  |
|  |  | The training materials successfully communicated the fundamental principles of the annual performance agreement. | LS3 |  |
|  |  | I received knowledge about the APA format, including its processes, measurement, and focal point function. | LS4 |  |
| C. | Behaviour | I have implemented the knowledge and skills acquired from the APA training in my professional duties within the organization. | BH1 | (Andhrutkar, 2019) |
|  |  | The APA training has positively influenced my work behaviour and practices. | BH2 |  |
|  |  | I have gained more confidence in carrying out activities linked to the annual performance agreement after the course. | BH3 |  |
|  |  | I possess knowledge of APA guidelines and understand my responsibilities following the completion of training. | BH4 |  |
| D. | Result | The APA training has contributed to improved overall performance in my role. | RE1 | (Alsalamah & Callinan, 2021; Andhrutkar, 2019) |
|  |  | The abilities acquired from the APA training have had a favorable influence on the results achieved by the organization. | RE2 |  |
|  |  | The APA training has been effective in achieving its intended goals. | RE3 |  |
|  |  | The APA is in accordance with the organization’s vision and objectives. | RE4 |  |
| E. | Effectiveness of APA Training | The APA training substantially enhanced my understanding of performance management ideas. | EAPA1 | (Bernardino & Curado, 2020; Mohamed & Alias, 2012) |
|  |  | The training materials effectively conveyed the essential elements of the annual performance agreement. | EAPA2 |  |
|  |  | The APA training positively influenced my ability to set and achieve performance goals. | EAPA3 |  |
|  |  | I have observed the positive impact on the organisation's overall results due to the APA training. | EAPA4 |  |
|  |  | I believe the APA training has prepared me well for handling performance-related responsibilities. | EAPA5 |  |

Alsalamah, A., & Callinan, C. (2021). Adaptation of Kirkpatrick’s four-level model of training criteria to evaluate training programmes for head teachers. *Education Sciences, 11*(3), 116.

Andhrutkar, R. (2019). A study of impact of behavioural training on individual performance measured through Kirkpatrick model for select software firms in pune. *Indira Management Review, 13*, 69-85.

Bernardino, G., & Curado, C. (2020). Training evaluation: a configurational analysis of success and failure of trainers and trainees. *European Journal of Training and Development, 44*(4/5), 531-546. doi:10.1108/EJTD-10-2019-0177

Hsu, W., & Chen, P.-W. (2021). The influences of service quality and individual characteristics on vocational training effectiveness. *Sustainability, 13*(23), 13207.

Mohamed, R., & Alias, A. A. S. (2012). *Evaluating the effectiveness of a training program using the four level Kirkpatrick model in the banking sector in Malaysia*. Paper presented at the 12-13 March, 3rd International Conference on Business and Economic Research, Bandung, Indonesia. <http://library.oum.edu.my/repository/id/eprint/717>
